# Observations of Deep Current at the Western Boundary of the Northern Philippine Basin

**DOI:** 10.1038/s41598-018-32541-9

**Published:** 2018-09-25

**Authors:** Chun Zhou, Wei Zhao, Jiwei Tian, Qingxuan Yang, Xiaodong Huang, Zhiwei Zhang, Tangdong Qu

**Affiliations:** 1 Key Laboratory of Physical Oceanography/CIMST, Ocean University of China and Qingdao National Laboratory for Marine Science and Technology, Qingdao, 266100 China; 2 Joint Institute for Regional Earth System Science and Engineering, University of California, Los Angeles, CA 90095 USA; 3 Key Laboratory of Marine Science and Numerical Modeling, First Institute of Oceanography, State Oceanic Administration, Qingdao, 266000 China

## Abstract

One-year time series of current velocities and hydrographic parameters based on four deep moorings deployed east of the Luzon Strait are employed to study the deep current at the western boundary (DCWB) of the northern Philippine Basin. While the mean current is relatively weak, the DCWB is highly variable on an intraseasonal time scale, with dominant periods ranging between 30 and 80 days. During the period of observation (October 2011–October 2012), the DCWB reversed its direction at early April, and pointed southward (−2.4 cm/s) in summer/autumn and northward (1.7 cm/s) in winter/spring. This annual reversal of the DCWB is consistent with the water property distribution in the deep Philippine Basin, with relatively cold and fresh water to the north and relatively warm and salty water to the south. The moored time series also allow for discussion on the stratification of the deep Luzon Strait, which indicates the lower interface of Pacific deep water capable of furnishing the deepwater overflow in the Luzon Strait.

## Introduction

As the only connection between the deep Pacific and South China Sea (SCS), deepwater overflow through the Luzon Strait has been intensively investigated recently^1–5^ for its potentially important role in generating the SCS deep circulation (SCSDC) and the regional climate^6^. While significant advances have been achieved in understanding the spatio-temporal structure of the overflow, its dynamics and possible role in regulating regional thermohaline circulation are less known. Several studies have investigated the SCSDC based on climatological databases^1,7^, model simulations^8–13^, and mooring observations^14^. This study focuses on the upstream of the Luzon Strait deep water overflow, i.e., the deep current at the western boundary (DCWB) of the northern Philippine Basin.

**Figure 1:**
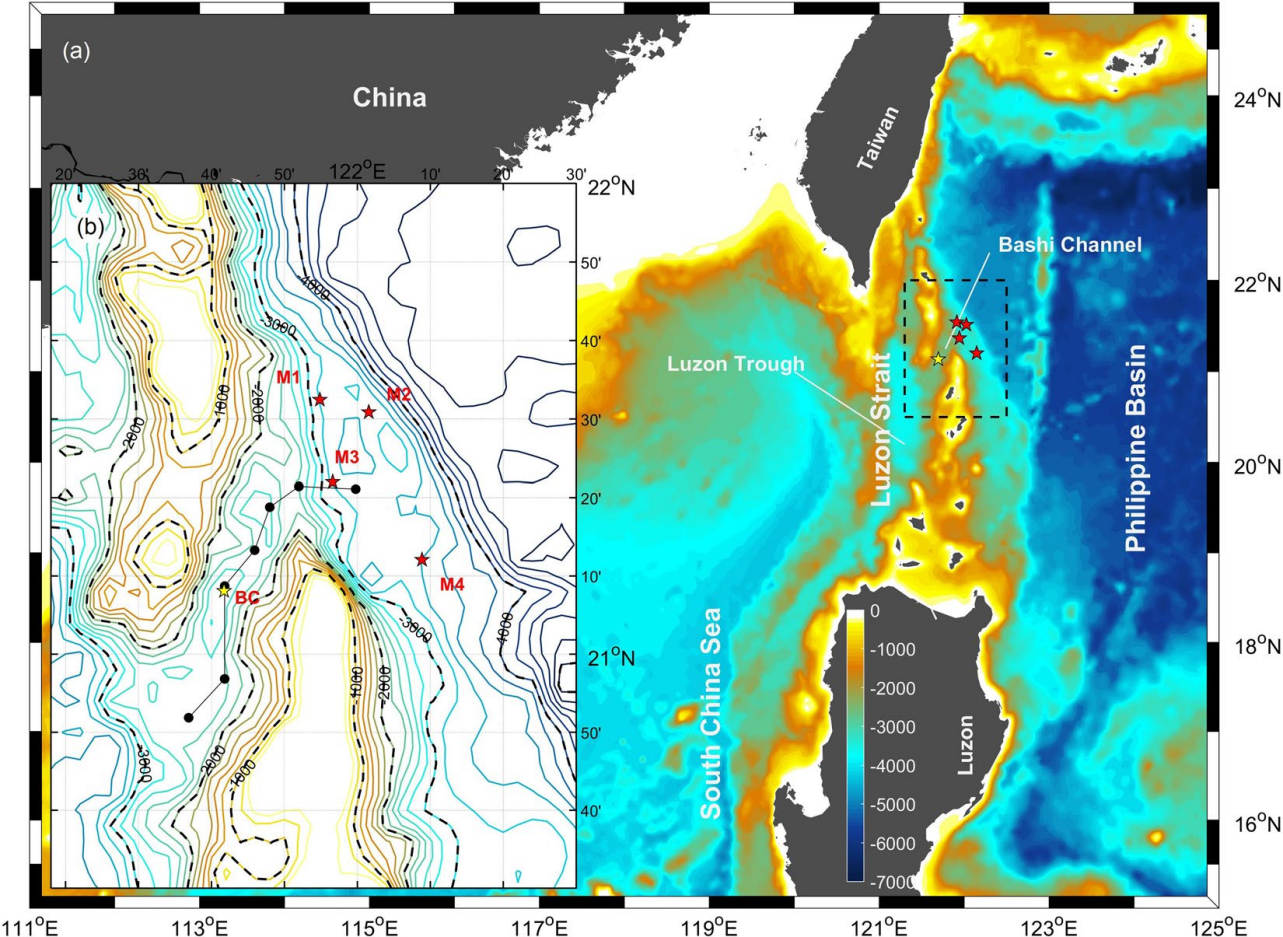
Bathymetry of the northwestern Philippine Basin and Luzon Strait^34^, with zoom-in plot of the topography in the box marked by dashed lines. Mooring locations of M1–M4 and BC are indicated in red and yellow pentagrams, respectively. Black dots connected by black solid line indicate the CTD section. Bathymetry data are downloaded from http://topex.ucsd.edu/marine_topo/. Figures are plotted using MATLAB R2016b (http://www.mathworks.com/) with M_Map (a mapping package, http://www.eos.ubc.ca/~rich/map.html).

The western boundary current of the Philippine Basin has been discussed by a series of studies over the past few decades^15–19^. However, with most of these earlier studies focused on the upper and intermediate layers, the DCWB of the northern Philippine Basin was barely investigated. The only relevant observational study was based on three moorings located east of the Luzon Strait^20^. It was claimed that a clear deep boundary current does not exist at the western boundary of the northern Philippine Basin, and the abyssal current there is unstable, which has also been reported by observations from abyssal current meters at the western boundary of the southern Philippine Basin^21^. Until now, the spatio-temporal characteristics of the DCWB of the northern Philippine Basin and the connection of the DCWB to the deepwater overflow in the Luzon Strait have never been examined. To address this issue, four deep moorings were deployed east of the Luzon Strait (M1–M4, Fig. 1) from October 2011 to October 2012. The results of these measurements are reported in this study.

## Basics of the DCWB

**Figure 2:**
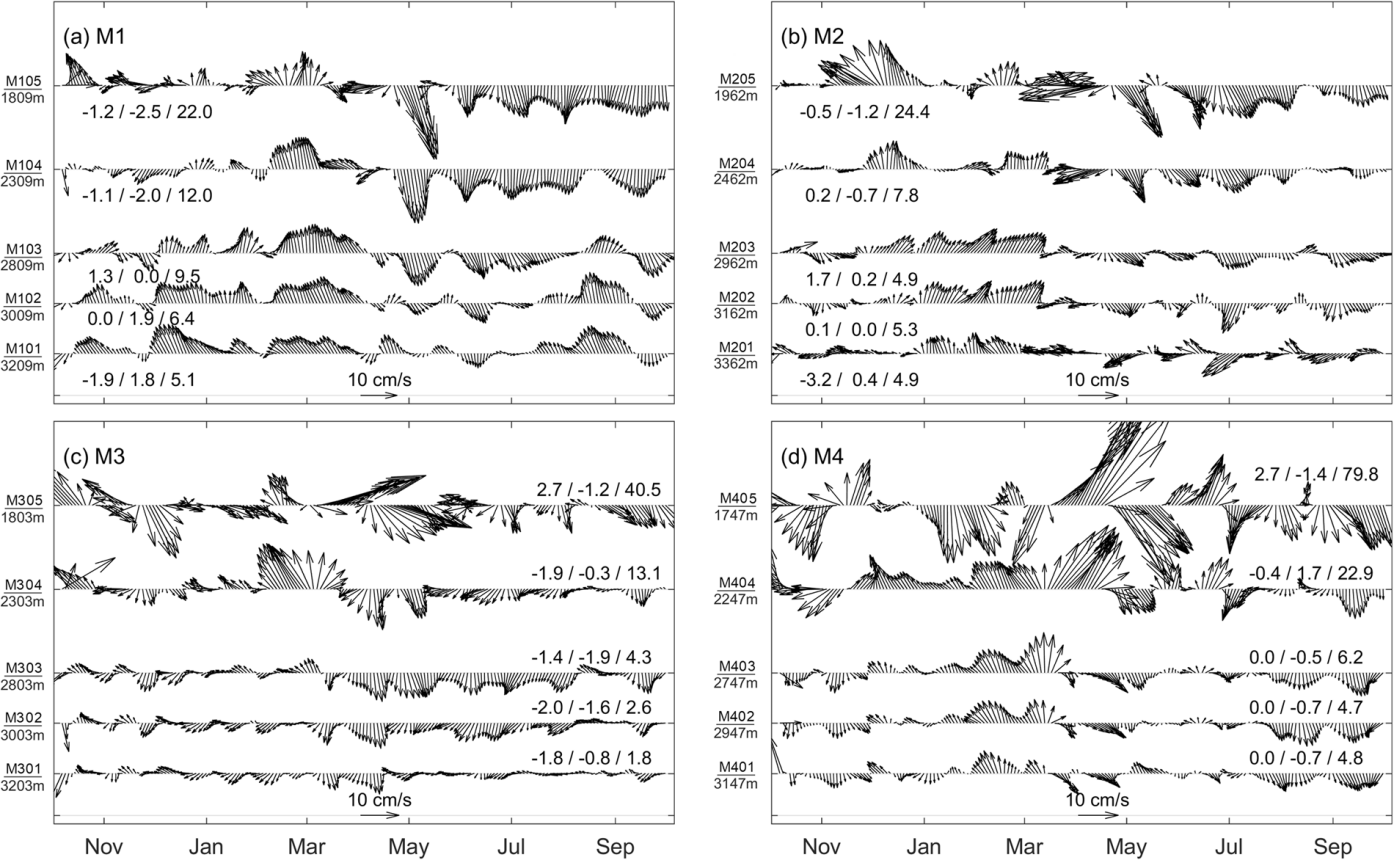
Vectors of the 15-day low-passed velocities at each observed depth of M1–M4. The mean U, V components (cm/s) and EKE calculated based on low-passed velocities (cm/s, in the brackets) are also indicated. The labels of y-axes indicate the names and depths of current meters. Figures are plotted using MATLAB R2016b (http://www.mathworks.com/).

Based on time series of the zonal (U) and meridional (V) velocity components at M1–M4 (Fig. S1), significant tidal signals are recorded for each layer. Focusing on the subinertial signals, a 15-day Butterworth low-pass filter is applied to the original velocity time series (Fig. 2). Due to the constraint of the deep western boundary, the V components at the four stations are found to be much stronger than the U components, with a vertically averaged mean ratio between the two components exceeding 5.0 at M1–M4. Hereafter, we focus on the V components of the DCWB unless otherwise specified.

The annual mean velocities of the deep boundary current are shown by averaging the low-passed velocity time series in each layer at M1–M4 (Fig. 2). The vertical mean V components are close to 0 cm/s at M1, M2 and M4, which suggests that there is no substantial DCWB there. This result is consistent with the results presented in a previous study^20^. At M3, which is located east of the mouth of the Bashi Channel, the mean current is also weak, but it flows in the same direction in all five layers, which is consistent with the intrusion of North Pacific deep water into the Bashi Channel.

**Figure 3:**
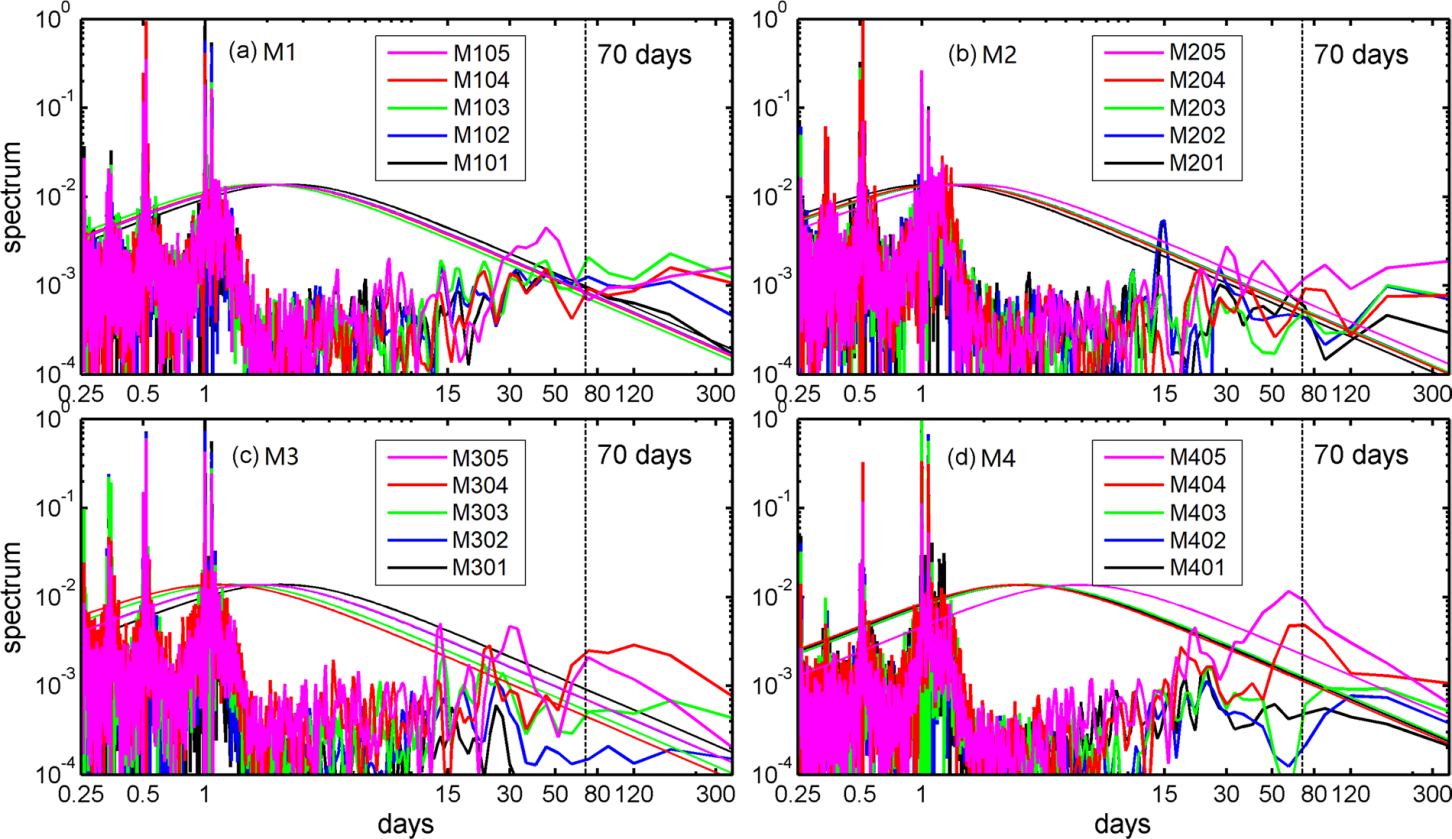
Spectrum analysis of the V components at M1–M4. The smooth lines indicate the 95% confidence level of the spectrum curves. Figures are plotted using MATLAB R2016b (http://www.mathworks.com/).

Despite the weak annual mean current, strong variability is seen in Fig. 2. Eddy kinetic energy (EKE) increases upward from the ocean bottom (Fig. 2), implying energetic variability in the intermediate and possibly upper layers^19^. Quiver plots of the low-passed velocity series display energetic subinertial variability with periods ranging from one to three months. The spectrum analysis of the original velocity series (Fig. 3) indicates that a characteristic period of ~30–80 days dominates variability at M1–M4, which was also noted for the Luzon Undercurrent at a depth ranging from 400 m to 700 m^19,22^. At M3, variability within a period of ~20–30 days appears to be notably energetic from 1800 m to 2800 m, which is similar to the intraseasonal variability found in the Bashi Channel^4^. Further discussion will be found in Section 4.

## Annual Reversal

**Figure 4:**
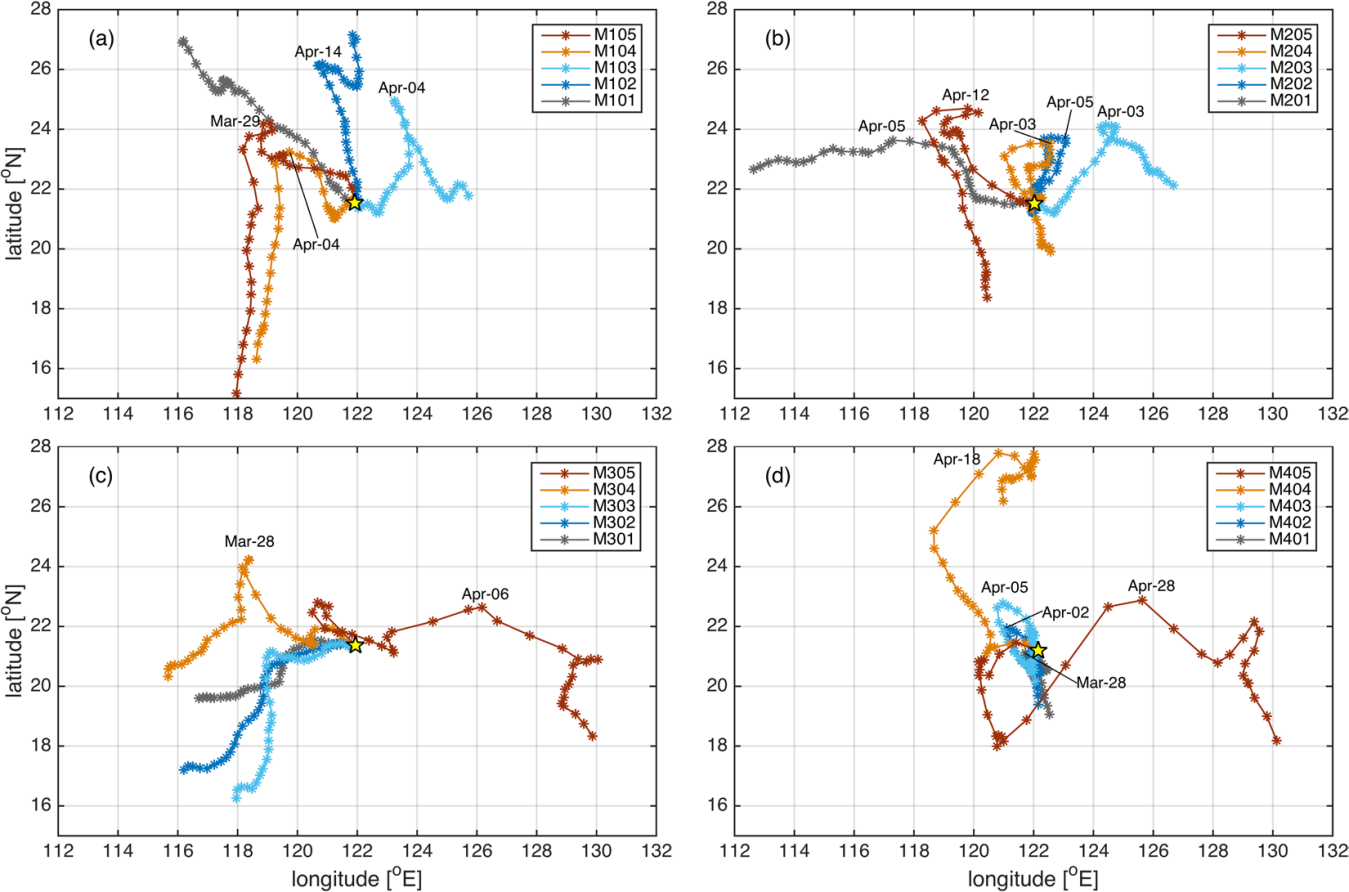
Progressive vector diagrams of the current at different depths of M1–M4 (panel a-d) based on daily velocity time series. Yellow pentagrams indicate the original positions. The colored lines indicate the results calculated from the different velocity time series at different depths. Elapsed times are marked every 10 days by asterisks. Timing of reversal happened are also indicated. Depths of each layer labeled in the panels are indicated in Fig. 2. Figures are plotted using MATLAB R2016b (http://www.mathworks.com/).

Sediment provenance and transport processes have been useful tools when tracing the origin of deep water. It has been speculated that there is a southward deep current that transports suspended sediments from the southeast coast of Taiwan to the deep Bashi Channel^23,24^. However, most of the dataset, including our measurements and those from previous studies^20^, does not show a steady DCWB east of the Luzon Strait. Careful examination of Fig. 2 indicates that the DCWB east of the Luzon Strait changes its direction at early April. Despite the energetic intraseasonal variability, the V component at M1, M2 and M4 generally points northward from October 2011–April 2012 (winter/spring) and reverses from May–October 2012 (summer/autumn). To better describe the annual reversal of the DCWB, progressive vector diagrams are employed (Fig. 4). The annual reversal can be identified in the four upper layers, except at M3 and the top layer at M4, which will be discussed in Section 4. Virtual displacements are directionally stable during each period and can be achieved at up to 800 km (e.g., M1 in summer/autumn). Exceptions with short period anomalies can be found due to energetic mesoscale processes, such as those during August 2012 at M1 below 3000 m. At M3, the flow is southwestward all year, which is presumably related to the bottom topography and deepwater overflow in the Bashi Channel.

**Figure 5:**
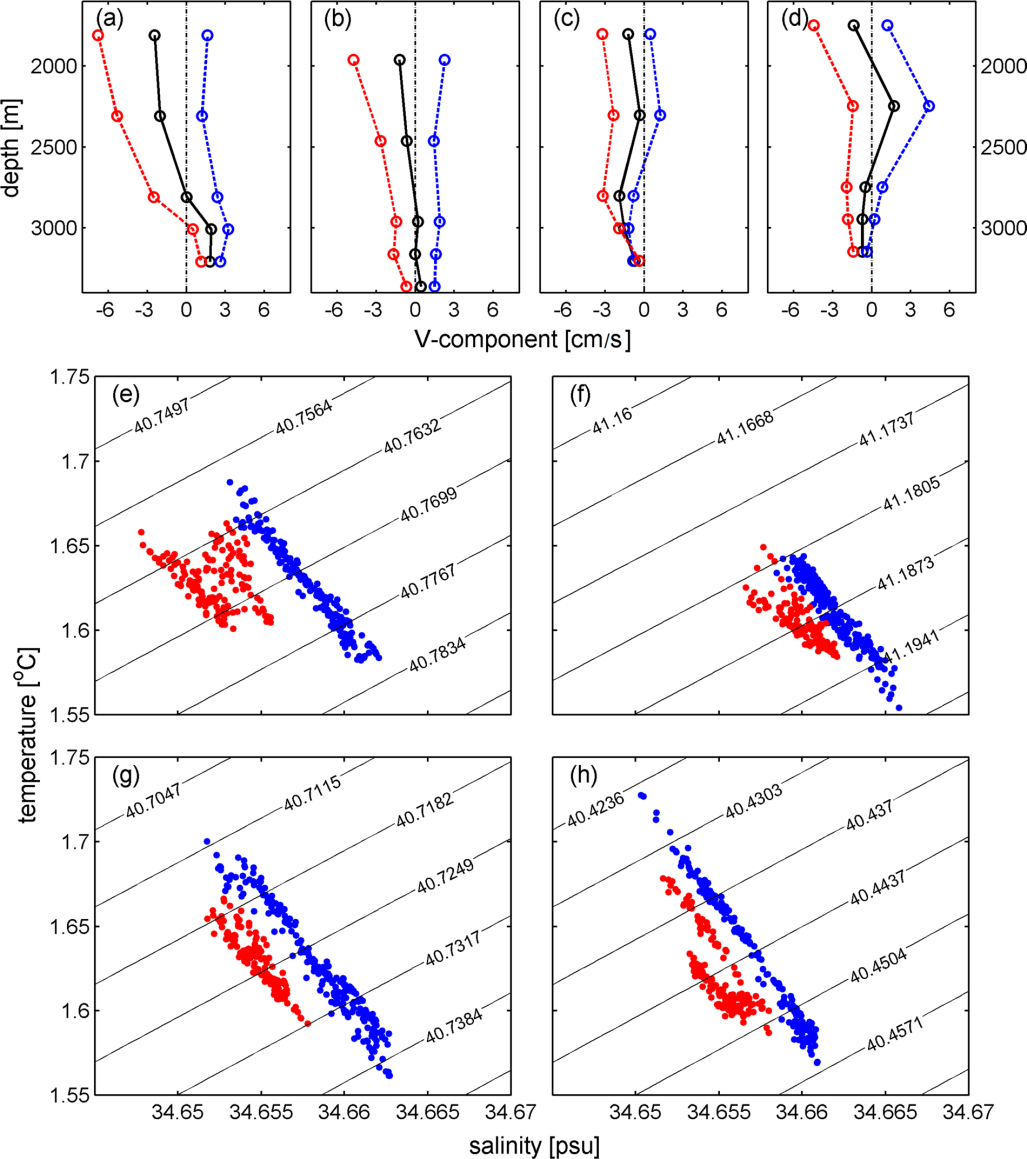
Panel a–d: Mean velocity profiles of V at M1–M4, respectively. Black, blue, and red lines stand for the mean profiles of the whole year, before and after April, respectively. Mean velocities at the observed depths are indicated by circles. Panel e-h: T/S diagrams based on daily mean temperature and salinity time series at ~2800 m of M1–M4. Blue (red) dots indicate the data from October 2011–April 2012 (May 2012–October 2012). Figures are plotted using MATLAB R2016b (http://www.mathworks.com/).

Mean vertical profiles of the V component during winter/spring and summer/autumn are shown in Fig. 5a–d. The velocities derived from these profiles at M1, M2 and M4 are 1.7 cm/s during winter/spring and −2.4 cm/s during summer/autumn, indicating a slightly stronger southward deep boundary current in summer/autumn than the northward deep boundary current in winter/spring. The velocity in summer/autumn decreases with depth below 1800 m, which is not observed in winter/spring (Fig. 5a–d). Therefore, the differences in velocity between the two periods tend to decrease with depth. At a depth of 2000 m, the differences at M1, M2 and M4 reach 6.8 cm/s, while they decrease to 2.6 cm/s at 3000 m.

If the annual reversal of the DCWB is robust, there must be a hydrographic signature due to different origins of water masses in winter/spring and summer/autumn. The Conductivity-Temperature-Depths (CTDs) mounted on the four moorings at ~2800 m allow us to confirm this conjecture. Similar to what has been done with the velocity time series, the temperature and salinity time series are also low-passed with a window longer than 15 days. T/S diagrams are presented to verify the annual reversal of the DCWB (Fig. 5e,f). By marking the T/S dots for winter/spring and summer/autumn with different colors, a notable difference between them stands out, indicating that the water of the deep boundary current is warmer and saltier in winter/spring than that in summer/autumn. The mean temperature (salinity) values based on the low-passed time series of CTDs of M1–M4 for these two periods are 1.624 °C (34.659 psu) and 1.621 °C (34.655 psu), respectively. Although partially compensated by the variability in temperature, the variability in potential density (σ_2_) is dominated by that in salinity, with a slightly higher potential density (36.977 kg/m^3^) during winter/spring than that during summer/autumn (34.975 kg/m^3^). This difference in the T/S relationship indicates different origins of deep water between the two periods, which corresponds well with the annual reversal of the DCWB.

**Figure 6:**
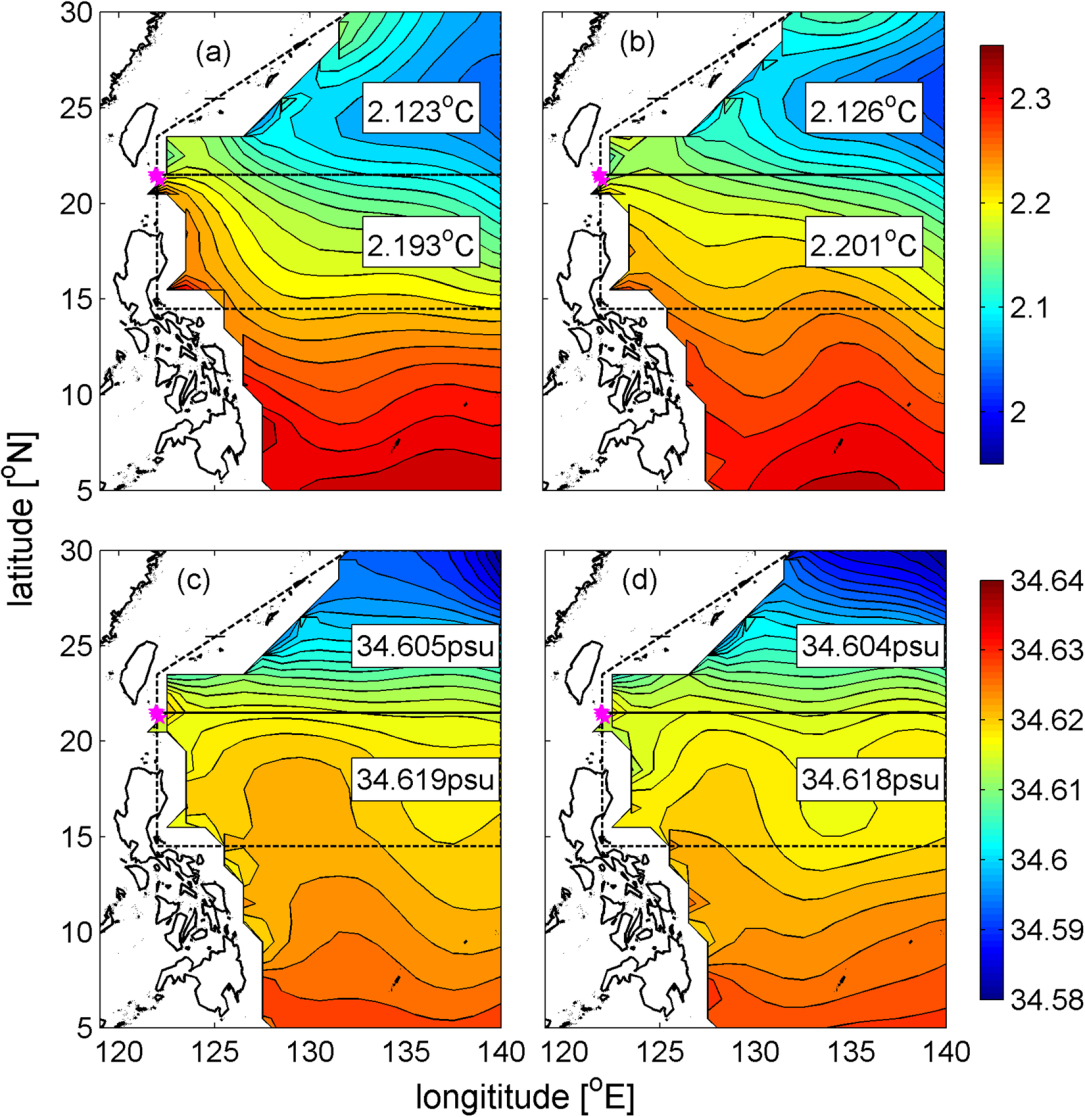
Temperature (upper panels) and salinity (lower panels) distribution in winter (left panels) and summer (right panels) at 2000 m in the Philippine Basin. Mooring locations of M1–M4 are indicated in magenta pentagrams. The northern Philippine Basin is divided into two subdivisions, with the horizontal mean values indicated in the white boxes. Figures are plotted using MATLAB R2016b (http://www.mathworks.com/).

To further illustrate the variability in the T/S relationship, the temperature and salinity distributions in the deep western Philippine Basin from the World Ocean Atlas 2013 (WOA13) are presented in Fig. 6. Since the reversal is observed at the whole water column below 1800 m, the temperature and salinity at 2000 m, which is the maximum depth of most Argo observations, are presented here. Substantial latitudinal gradients are seen in the temperature and salinity fields, implying that the southward deep boundary current in summer/autumn carries relatively cold and fresh water from the higher latitudes of the Philippine Basin, while the northward deep boundary current in winter/spring transports relatively warm and salty water from the lower latitudes of the Philippine Basin. Note that the temperature and salinity off the western boundary also vary on a seasonal time scale. If the deep Philippine Basin becomes colder in winter, water carried by the DCWB should also become colder. By calculating the mean temperature and salinity in summer and winter in the two subregions of the northern Philippine Basin (Fig. 6), we find that the seasonal variabilities in temperature and salinity are minor and negligible compared to the spatial variability due to meridional gradient, which indicates that variability in the water properties of the DCWB is mainly related to meridional temperature and salinity gradients. However, it should be noted that the limited samples of the WOA13 in each grid square of the deep Philippine Basin (Fig. S2) may somehow influence the validity of the temperature and salinity distributions and their seasonal variabilities.

## Connection to Deepwater Overflow in the Bashi Channel

As mentioned above, the DCWB is found to point northward in winter/spring and southward in summer/autumn, with exceptions at M3 and the top layer at M4 (Fig. 5). A roughly eastward current dominates the top layers at M3 and M4 (Fig. 4). This depth (~1800 m) lies in the intermediate layer of the Luzon Strait (~500–2000 m), which has been reported to have an eastward net transport by several previous studies^8,25–27^. A recent study based on continuous observations of approximately one year via a mooring array depicted the complex structure of zonal flow in the intermediate layer^28^. An eastward flow was indicated to the north of 21.1°N, which is consistent with the eastward displacement in the top layer at M3 and M4.

**Figure 7:**
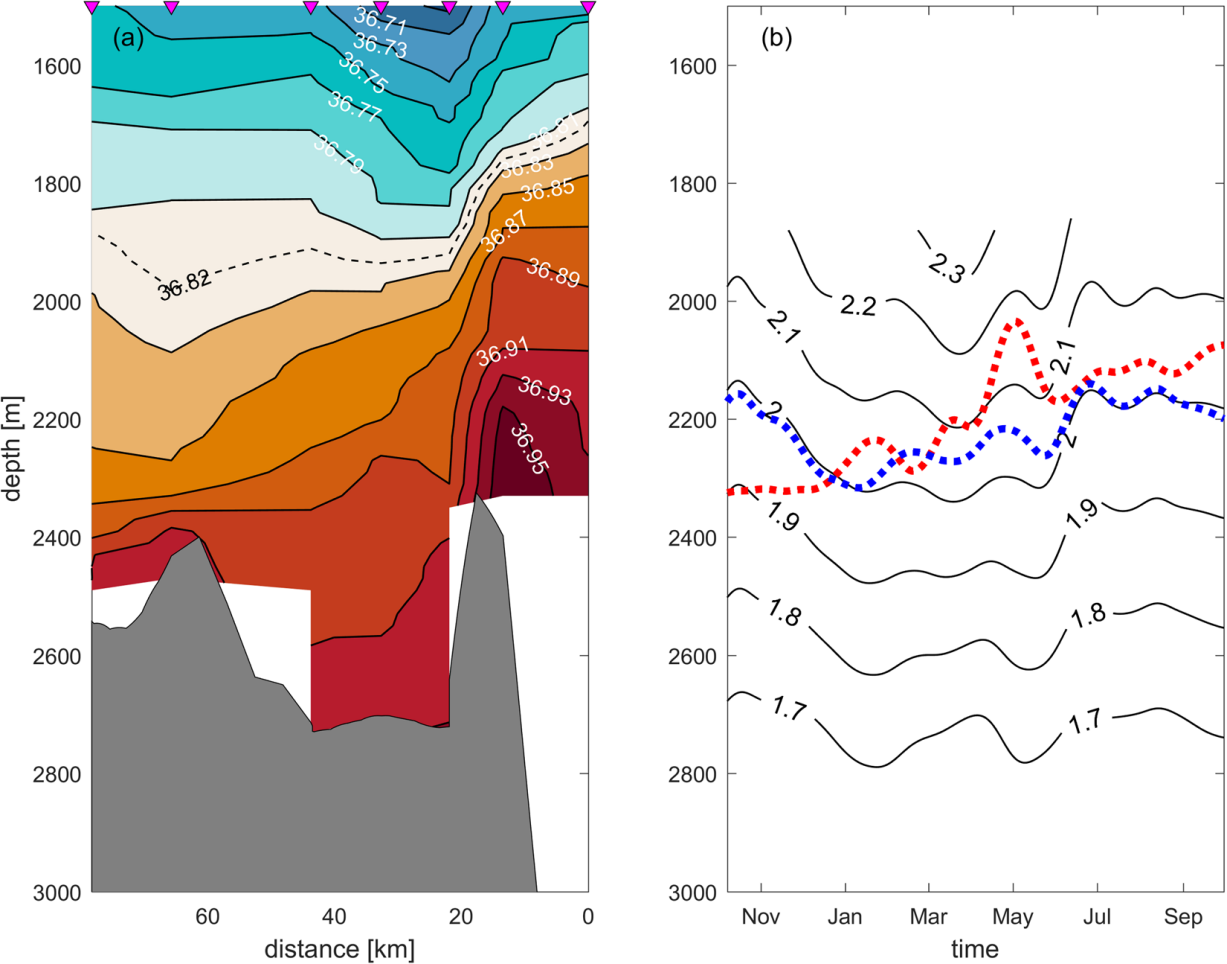
Panel a: Sectional view of the potential density (σ_2_) based on CTD casts along the Bashi Channel shown in Fig. 1b; Panel b: Determining the depth of lower interface of deep water spilling into the Bashi Channel. Black solid lines indicate the temperature contours at M3. Red dash lines indicates the temperature at 120 m above the bottom of BC from October 2011–October 2012. Blue dash lines indicates the mean temperature cycle at 120 m above the bottom of BC from October 2009–April 2013. Figures are plotted using MATLAB R2016b (http://www.mathworks.com/).

Due to the persistent pressure gradient between the northwestern Pacific and SCS, north Pacific deep water continuously spills into the deep Bashi Channel, and finally flows into the deep SCS, as indicated both by mooring observations^2,4,5^ and also Lowered-ADCP profiles^3^. Generally, southwestward flows are shown in the four lower layers of M3 (Fig. 5), also suggesting the continuous supply of cold and saline deep water to the Bashi Channel. Previous studies have chosen σ_2_ = 36.82 kg/m^3^ or roughly a 2000 m depth as the upper interface of deep water spilling into the deep Bashi Channel^3,4,6^. However, the lower interface of deep water spilling into the Bashi Channel at the western boundary of the Philippine Basin is not known. Based on the CTD casts sampled in 2008 along the Bashi Channel (Fig. 1b), section view of the stratification from the western boundary of the deep Philippine Basin to the Bashi Channel is presented (Fig. 7a). The elevation of upstream isopycnals, tilt when approaching the sill, and steepening after crossing the sill all imply a typical deep overflow^29,30^. Without a notable density difference between the two sides, σ_2_ = 36.79 kg/m^3^ can be a reasonable upper interface for the penetration. For the lower interface, σ_2_ = 36.93 kg/m^3^ is the densest isopycnal found in the Bashi Channel, which corresponds to an upstream depth range of ~2120–2260 m.

However, energetic temporal variability near the Luzon Strait^4^ could increase the uncertainty of the above results derived from snapshot observations of the CTD casts. Continuous observations from M3 and BC (which is a deep mooring in the Bashi Channel; see Section 6 for details) are employed here to provide evidence on the variability of the lower interface of deep water penetrating into the Bashi Channel. We linearly interpolate the temperature between M305 and M303, and determine the depth where the temperature equals that of the BC (red dashed line in Fig. 7b), which is then identified as the lower interface. The lower interface varies seasonally, with relatively deeper depths around winter and shallower depths around summer. However, cycle of the variability seems to be unclosed due to the interannual variability’s interruption on the seasonal cycle of temperature of the deepwater overflow in the Bashi Channel^4^. Therefore, based on continuous observations of the deep Bashi Channel from October 2009-April 2013, we estimate the mean seasonal cycle of temperature in the deep Bashi Channel and find the corresponding lower interface located upstream (blue dashed line in Fig. 7b). The lower interface basically follows the 2 °C isotherm (with an exception in spring) and remains deeper in winter/spring and shallower in summer/autumn between depth range of 2150–2320 m, which represents the densest water in the Philippine Basin capable of directly impacting the deep Luzon Strait and SCS. Considering that entrainment exists between the sill and BC due to enhanced mixing^31,32^ and that the CTD on mooring BC was mounted 120 m above the bottom, the densest water spills into the Bashi Channel should be somewhat colder than those observed at BC, which implies that the upstream interface should be a little deeper than shown in Fig. 7b.

As mentioned in Section 2, energetic variability with similar periods to that found in the Bashi Channel was observed at M3. To investigate the propagation direction of the variability signal, the hydraulic state should be estimated, which could be described by the Froude number ${\rm{F}}={\rm{V}}\,/\,\sqrt{g^{\prime} D}$, while V, g′, D stands for the layer-averaged flow velocity, reduced gravity, and layer depth, respectively. By averaging the velocity at different layers at M3, V is estimated to be 0.6 cm/s. ${\rm{g}}^{\prime} ={\rm{g}}\frac{{\rm{\Delta }}\rho }{\rho }$ could be calculated based on the CTD cast near M3. With isopycnal of 36.79 kg/m^3^ as the upper interface, F is estimated to be 0.01, which is much less than 1, suggesting that the flow is subcritical^33^ and variability signal should propagate against the stream from the deep Bashi Channel to M3. Considering the subcritical state also at the deep Bashi Channel^5^ and the propagation of intraseasonal variability against the stream in the northern Luzon Trough^4^, the intraseasonal variability could generated around the chock point of the deep Luzon Trough or even to the south and propagate all the way to the east of the Bashi Channel.

## Conclusions

The DCWB of the northern Philippine Basin is expected to play a role in connecting the deep Philippine Basin and the deepwater overflow in the Luzon Strait. Based on one-year observations of four deep moorings, this study has investigated the spatio-temporal characteristics of the DCWB. The main results are summarized as follows:

1. The annual mean DCWB of the northern Philippine Basin is fairly weak, suggesting that a typical, steady deep western boundary current does not exist outside the mouth of the Bashi Channel. However, energetic intraseasonal variability is observed in the DCWB, with dominant periods ranging between 30 and 80 days.
2. The DCWB reverses its direction in April, with a southward flow (−2.4 cm/s) in summer/autumn and a northward flow (1.7 cm/s) in winter/spring. Due to the latitudinal gradients of temperature and salinity in the deep Philippine Basin, the southward flow carries colder and fresher water than the northward flow.
3. Deep water of the Philippine Basin penetrates into the Bashi Channel, with a lower interface of ~2150–2320 m, which becomes deeper in winter/spring and shallower in summer/fall. With its connection to the deepwater overflow in the Luzon Strait, the penetration of deep Philippine Basin water could potentially impact the SCSDC.

Whereas new knowledge on the DCWB of the northern Philippine Basin and its connection to the deepwater overflow in the Luzon Strait is presented here, the limited duration and coverage of the mooring observations could result in uncertainty to some extent. Further observations would be helpful in enhancing the knowledge on the DCWB of the Philippine Basin.

## Data and Methods

Four moorings were positioned outside the mouth of the Bashi Channel, with two slightly to the north, one at the mouth, and one slightly to the south of the channel (Fig. 1). Each mooring was mounted with one NORTEK Deep Water Aquadopp current meter on the top and four Aanderaa RCM Seaguard current meters below it, which recorded the current velocity from approximately 1800 m to approximately 3200 m (120 m above the bottom). Each NORTEK Deep Water Aquadopp current meter was also assembled with a temperature and pressure sensor. Additionally, a Sea-Bird Electronics SBE 37-SM CTD was also configured onto each mooring at 5 m below the middle current meter (near 2800 m), to record the hydrographic characteristics of deep water. Deployed in October 2011 and recovered in October 2012, these moorings, including 20 current meters and 4 CTDs, yielded satisfactory current, temperature and salinity records for one year. Current meters and CTDs had standard calibrations from the manufacturers. The accuracies of the sensors are 0.002 °C (0.1 °C) for temperature of CTD (NORTEK current meter), 0.003 mS/cm for conductivity, and 7 m (15 m) for pressure of CTD (Nortek). The accuracies of the velocity measurements were 1% of the reading or 0.5 cm/s for NORTEK Aquadopp and 1% of the reading or 0.15 cm/s for Aanderaa RCM Seaguard. All current meters and CTDs were configured to record data at a sample interval of 1 hour. According to the pressure record via the CTDs on the moorings, the vertical excursion of the instruments was less than 30 m for 96% of the records, suggesting that the moorings were relatively stable during the period of observation. Therefore, errors resulting from current meter motion are expected to be small and are neglected in the discussion.

To study the connection between the DCWB and the deepwater overflow in the Bashi Channel, mooring observations during October 2009–April 2013 in the deep Bashi Channel used in a previous study^4^ (i.e., BC) are also employed here, including current and temperature observations 120 m above the bottom. In addition, a series of CTD profiles sampled in 2008 are employed here to support the study. The CTD used was an Sea-Bird Electronics SBE 911 plus, which was calibrated before and after the cruise in the laboratory.

Moreover, to describe the water property distribution in the deep Philippine Basin, the seasonal climatologies of temperature and salinity from the WOA13 (http://www.nodc.noaa.gov/OC5/woa13/) are also used in this study.

## Electronic supplementary material

Supplementary Information

## Data Availability

The data that support the findings of this study are available from the corresponding author on reasonable request.
