# Supplementary material for: Observations of Deep Current at the Western Boundary of the Northern Philippine Basin
Source: Sci Rep. 2018 Sep 25;8:14334. doi: 10.1038/s41598-018-32541-9 (PMC6156581; doi:10.1038/s41598-018-32541-9)
Supplement: Supplementary file 1 — Supplementary Information [file 41598_2018_32541_MOESM1_ESM.docx]

**Supplementary Information**

Observations of Deep Current at the Western Boundary of the Northern Philippine Basin

Chun Zhou^1^, Wei Zhao^1^*, Jiwei Tian^1^, Qingxuan Yang^1^, Xiaodong Huang^1^, Zhiwei Zhang^1^ & Tangdong Qu^2, 3^

1. Key Laboratory of Physical Oceanography /CIMST, Ocean University of China and Qingdao National Laboratory for Marine Science and Technology, Qingdao 266100, China.

2. Joint Institute for Regional Earth System Science and Engineering, University of California, Los Angeles, CA 90095, USA.

3. Key Laboratory of Marine Science and Numerical Modeling, First Institute of Oceanography, State Oceanic Administration, Qingdao 266000, China.

*Corresponding author: Wei Zhao, Key Laboratory of Physical Oceanography /CIMST, Ocean University of China and Qingdao National Laboratory for Marine Science and Technology, Qingdao 266100, China. (weizhao@ouc.edu.cn)


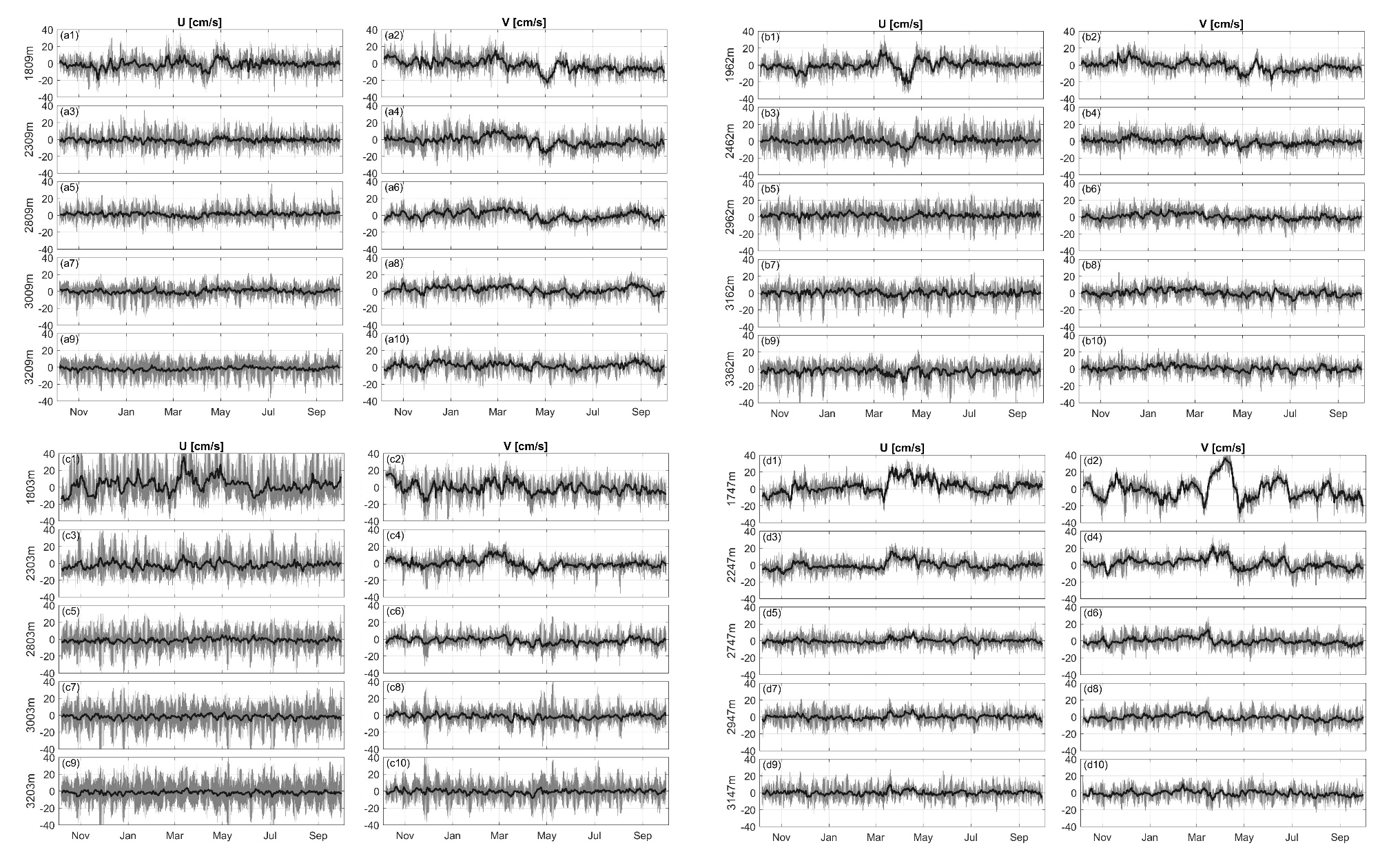


**Figure S1.** Velocity time series of U and V at M1 (panel a1~a10), M2 (panel b1~b10), M3 (panel c1~c10), M4 (panel d1~d10) at different depths. Depths are indicated as the labels of y-axes. Gray thin lines show the original time series, while black bold lines show the daily-mean time series. Figures are plotted using MATLAB R2016b (http://www.mathworks.com/).


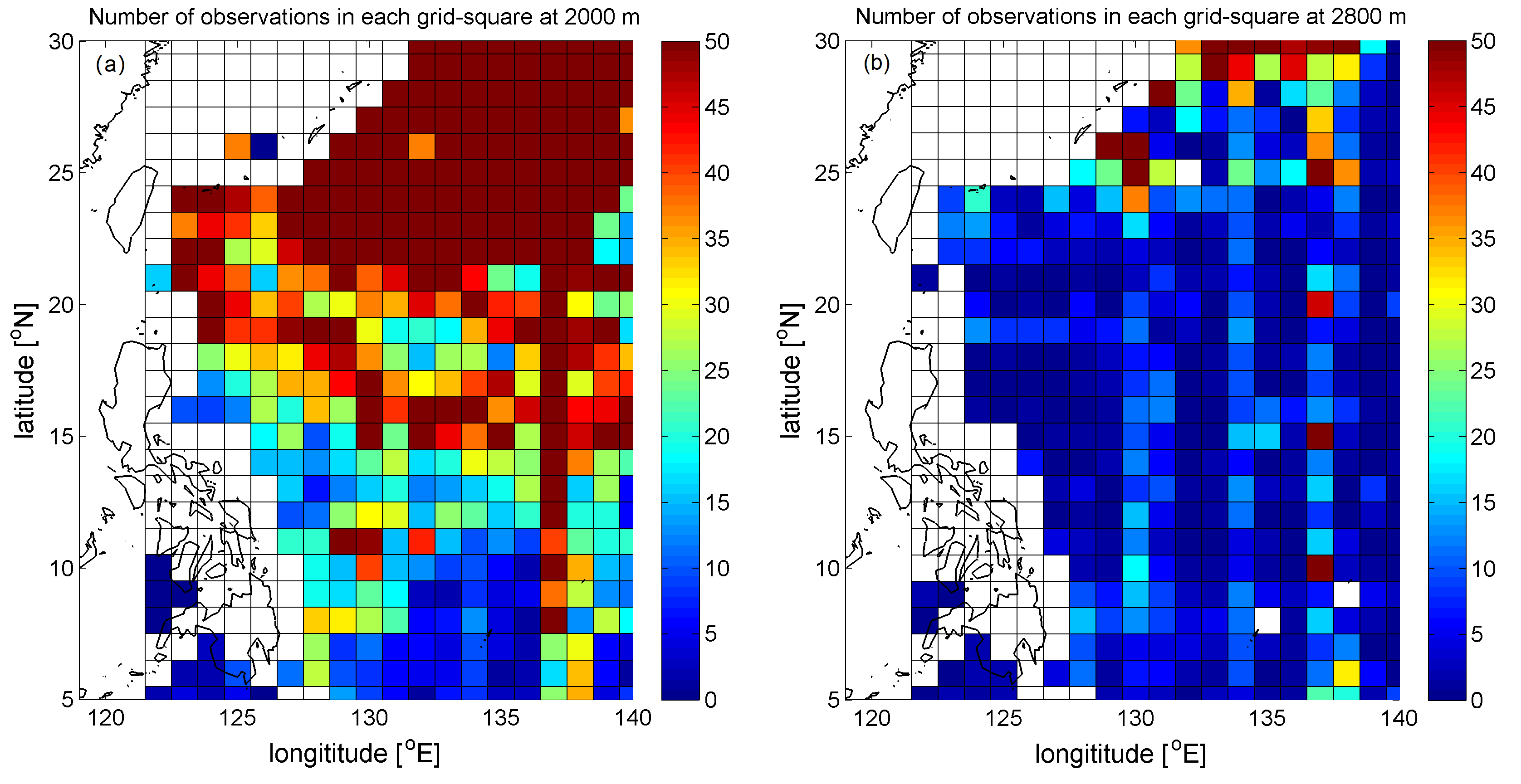


**Figure S2.** Total number of temperature and salinity observations in each grid-square at 2000 m (a) and 2800 m (b) based on the WOA13 seasonal climatology dataset. Figures are plotted using MATLAB R2016b (http://www.mathworks.com/)
